# WS_2_/Si_3_N_4_-Based Biosensor for Low-Concentration Coronavirus Detection

**DOI:** 10.3390/mi16020128

**Published:** 2025-01-23

**Authors:** Talia Tene, Fabian Arias Arias, Karina I. Paredes-Páliz, Ana M. Cunachi Pillajo, Ana Gabriela Flores Huilcapi, Luis Santiago Carrera Almendariz, Stefano Bellucci

**Affiliations:** 1 Department of Chemistry, Universidad Técnica Particular de Loja, Loja 110160, Ecuador; 2 Dipartimento di Chimica e Tecnologie Chimiche, University of Calabria, Via P. Bucci, Cubo 15D, 87036 Rende, Italy; 3 Grupo de Investigación en Salud Pública, Facultad de Ciencias de la Salud, Universidad Nacional de Chimborazo, Riobamba 060108, Ecuador; 4 Laboratorio Ciencias Biológicas, Facultad Recursos Naturales, Escuela Superior Politécnica de Chimborazo (ESPOCH), Riobamba 060155, Ecuador; 5 Facultad de Ciencias, Escuela Superior Politécnica de Chimborazo (ESPOCH), Riobamba 060155, Ecuador; 6 INFN-Laboratori Nazionali di Frascati, Via E. Fermi 54, 00044 Frascati, Italy

**Keywords:** surface plasmon resonance, tungsten disulfide, silicon nitride, ssDNA, SARS-CoV-2, biosensor, TMM approach, Fresnel equations

## Abstract

This study presents the optimization of two SPR biosensors, Sys_3_ and Sys_5_, for SARS-CoV-2 detection at concentrations of 0.01–100 nM. Sys_3_, with a 55 nm silver layer, a 13 nm silicon nitride layer, and a 10 nm ssDNA layer, achieved a figure of merit (FoM) of 571.24 RIU^−1^, a signal-to-noise ratio (SNR) of 0.12, and a detection accuracy (DA) of 48.93 × 10^−2^. Sys_5_, incorporating a 50 nm silver layer, a 10 nm silicon nitride layer, a 10 nm ssDNA layer, and a 1.6 nm tungsten disulfide layer (L = 2), demonstrated a higher sensitivity of 305.33 °/RIU and a lower limit of detection (LoD) of 1.65 × 10^−5^. Sys_3_ outshined in precision with low attenuation (<1%), while Sys_5_ provided enhanced sensitivity and lower detection limits, crucial for early-stage viral detection. These configurations align with the refractive index ranges of clinical SARS-CoV-2 samples, showcasing their diagnostic potential. Future work will focus on experimental validation and integration into point-of-care platforms.

## 1. Introduction

The emergence of Severe Acute Respiratory Syndrome Coronavirus 2 (SARS-CoV-2) in late 2019 has triggered one of the most significant global health crises in modern history [1]. SARS-CoV-2, the causative agent of the COVID-19 pandemic, has resulted in substantial morbidity and mortality worldwide, while severely impacting healthcare systems, economies, and social structures [2]. The virus, a member of the Coronaviridae family, is characterized by its rapid human-to-human transmission and its ability to cause severe respiratory illness, particularly in vulnerable populations [3]. Despite the development of vaccines and therapeutic interventions, effective diagnostic tools remain essential for managing the pandemic, enabling timely identification, isolation, and treatment of infected individuals.

Typical SARS-CoV-2 detection techniques include nucleic acid-based methods [4], such as reverse transcription–polymerase chain reaction (RT–PCR) [5], antigen-based assays [6], and serological tests [7]. While RT–PCR is considered the gold standard for its high sensitivity and specificity, it requires sophisticated laboratory infrastructure and trained personnel, making it less feasible for large-scale, rapid testing in resource-constrained settings. Antigen and serological tests offer faster results but often suffer from reduced accuracy, particularly in the early stages of infection. The limitations of these conventional methods have highlighted the need for innovative diagnostic technologies that combine high sensitivity, specificity, and real-time capabilities with ease of deployment.

In this context, surface plasmon resonance (SPR) biosensors have emerged as a promising alternative for SARS-CoV-2 detection [8]. SPR-based devices exploit the interaction of light with surface plasmons—coherent oscillations of free electrons at a metal-dielectric interface [9]—to achieve real-time, label-free detection of biomolecular interactions [10]. These sensors are highly sensitive to changes in the refractive index near the sensor surface [11], making them ideal for detecting viral proteins, nucleic acids, or antibodies with minimal sample preparation. Furthermore, SPR biosensors can be tailored for high-throughput applications, offering significant advantages over traditional diagnostic approaches. For instance, SPR-based platforms with tailored resonance shapes, such as dielectric gratings and box-like resonance configurations, have demonstrated significant sensitivity improvements by mitigating the spectral overlap challenges associated with Lorentzian shapes, as evidenced in ultra-compact photonic crystal-based sensors [12].

The theoretical foundation of SPR biosensors lies in the physics of surface plasmon resonance, governed by the interaction between electromagnetic waves and free electrons in metals such as gold or silver [13]. This interaction is described by the Fresnel equations [14], which characterize light reflection and transmission at layered interfaces, and by the transfer matrix method (TMM) [15], which models light propagation through complex multilayer structures. By understanding these principles, SPR biosensors can be optimized for maximum sensitivity and specificity, particularly when detecting low-concentration analytes like viral RNA or antigens. For instance, waveguide-based designs, such as bimodal configurations utilizing subwavelength grating structures, have shown exceptional refractive index sensitivities and robust detection limits, making them viable candidates for biosensing platforms [16].

Recent advances in materials science have further enhanced the performance of SPR biosensors through the integration of two-dimensional (2D) materials, such as graphene [17] and tungsten disulfide (WS_2_) [18]. These materials exhibit exceptional optical, electronic, and surface properties that synergistically improve the sensitivity and stability of SPR-based platforms. WS_2_, in particular, has gained attention for its high refractive index, strong excitonic resonances, and tunable optical properties, which enable efficient light-matter interaction at the nanometer scale [19]. Additionally, WS_2_ offers a chemically versatile surface for functionalizing biomolecules, enhancing the specificity of biosensors for SARS-CoV-2 detection [18]. Chirped guided-mode resonance biosensors, which incorporate advanced grating designs, further demonstrate the utility of such innovative approaches by achieving cost-effective, highly sensitive, and portable biosensing solutions [20].

Despite these advancements, there is a notable gap in the literature regarding the use of WS_2_ in SPR biosensors, particularly for detecting SARS-CoV-2 at very low concentrations (~10^9^ viral particles/mL or ~0.01 nM). While 2D materials have been extensively studied for their optical and electronic properties, their integration into SPR platforms for ultra-sensitive viral detection remains underexplored [21]. This lack of comprehensive studies limits the development of robust, optimized biosensing systems that can effectively address the challenges of low viral load detection in clinical samples. Therefore, it is crucial to investigate the potential of WS_2_-based SPR biosensors in this context, focusing on achieving high sensitivity and specificity through systematic layer optimization and performance evaluation.

Here, our study focuses on understanding the role of each layer in the proposed SPR biosensor, which is constructed using a BK7 glass substrate, a silver (Ag) film, a silicon nitride (Si_3_N_4_) layer, WS_2_, single-stranded DNA (ssDNA), and phosphate-buffered saline (PBS) solution as the surrounding medium. Each layer is systematically optimized to identify promising configurations that maximize sensor performance. The results are analyzed in the context of key metrics, including attenuation, full-width at half-maximum (FWHM), and sensitivity enhancement. Furthermore, the biosensor’s performance is evaluated by examining metrics such as sensitivity to refractive index changes, detection accuracy (DA), quality factor (QF), figure of merit (FoM), limit of detection (LoD), and signal-to-noise ratio (SNR). Unlike prior studies that focus on analyte concentrations ranging from 150 mM to 525 mM [22], which are unrealistic in clinical samples, our work specifically targets a clinically viable concentration range of 0.01 nM to 100 nM, making it more relevant for realistic diagnostic applications. These insights aim to contextualize the design and operation of advanced SPR biosensors for SARS-CoV-2 detection.

## 2. Materials and Methods

### 2.1. Initial Parameters and Configurations

**Figure 1:**
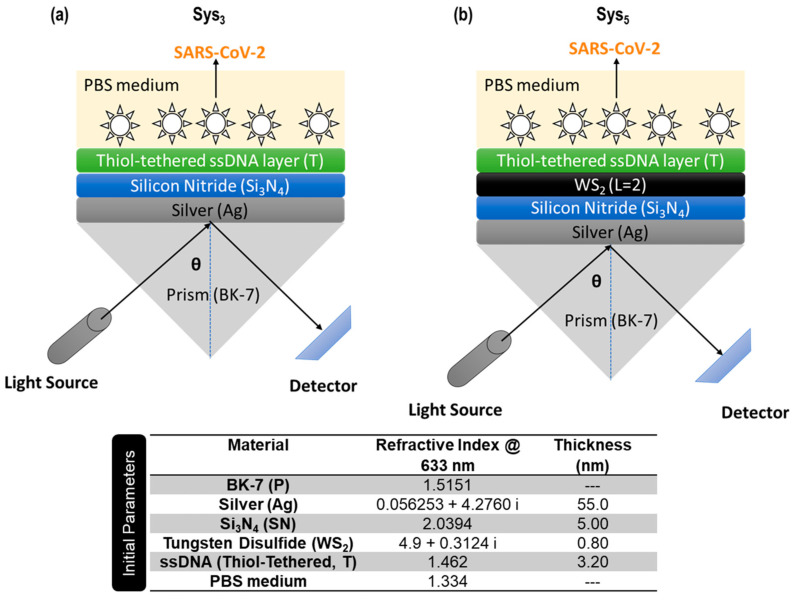
Schematic representation of the proposed biosensors against SARS-CoV-2, including the initial parameters at 633 nm for optimization. (**a**) Multilayer biosensor without WS_2_ layer and (**b**) Multilayer biosensor including WS_2_ layer.

The initial parameters of the SPR biosensor layers, including refractive indices at 633 nm and thicknesses, were selected based on previous experimental and theoretical results [18,21,22,23,24] (see Table S1 and Figure 1). The BK7 glass substrate, with a refractive index of 1.5151, was chosen for its optical clarity and compatibility with SPR systems. Its stability provides a robust platform for constructing the multilayer configuration. Silver (Ag), with a refractive index of 0.056253 + 4.2760 *i* and a thickness of 55 nm, was selected as the plasmonic material. A 5 nm silicon nitride (Si_3_N_4_) layer, with a refractive index of 2.0394, was incorporated to stabilize the silver layer and support the adhesion of subsequent layers. This dielectric material also plays a role in modulating the refractive index profile of the sensor [22]. A monolayer (0.8 nm thickness) of WS_2_, with a refractive index of 4.9 + 0.3124 *i*, was added to exploit its unique optical and electrical properties [25]. Particularly, WS_2_ is expected to enhance the plasmonic field confinement due to its high refractive index and strong excitonic interactions [26], which are critical for improving the sensor’s sensitivity. The functionalization layer, consisting of 3.2 nm of thiol-tethered single-stranded DNA (ssDNA) with a refractive index of 1.462, enables specific detection of SARS-CoV-2 RNA. This layer ensures selective binding to the target analyte while maintaining signal integrity [23]. The surrounding medium, modeled as phosphate-buffered saline (PBS) with a refractive index of 1.334, provides a physiological environment for biomolecular interactions [24].

Table 1 outlines the stepwise progression of SPR biosensor configurations, starting from a basic system (Sys_0_) to a fully functional and advanced structure (Sys_5_). This systematic approach allows us to evaluate each configuration under different environments (i.e., water and phosphate-buffered saline) to identify the most promising designs for further optimization. The baseline configuration, Sys_0_, consists of the prism, silver film, and water medium (P/Ag/M_H2O_). This setup provides the initial framework to study the intrinsic plasmonic response of the silver layer, which serves as the primary plasmonic material in all subsequent systems. The use of water as the medium ensures that the results are not influenced by complex refractive index effects, serving as a reference point for later comparisons. Sys_1_ introduces PBS as the medium, replacing water while maintaining the basic prism/silver structure (P/Ag/M_PBS_). This configuration reflects a transition toward more clinically relevant environments, as PBS closely mimics the conditions encountered in realistic diagnostics.

Sys_2_ builds on Sys_1_ by incorporating a silicon nitride layer between the silver film and the medium (P/Ag/SN/M_PBS_). In Sys_3_, a thiol-tethered ssDNA layer is added to the silicon nitride surface (P/Ag/SN/T/M_PBS_). Sys_4_ introduces a tungsten disulfide layer to the structure (P/Ag/SN/WS_2_/M_PBS)_. Finally, Sys_5_ combines all components (i.e., prism, silver, silicon nitride, tungsten disulfide, ssDNA, and PBS medium (P/Ag/SN/WS_2_/T/M_PBS_). The latter configuration represents the most advanced system under investigation, incorporating functional and structural elements for maximum sensitivity and specificity.

Figure 1 illustrates the two most promising SPR biosensor configurations, Sys_3_ and Sys_5_, which demonstrate the progression from a baseline multilayer design to an advanced structure incorporating tungsten disulfide. While the results justifying their selection will be discussed in the next section, this figure provides a clear visualization of the proposed multilayer architecture. In particular, Figure 1a depicts the Sys_3_ configuration, which integrates a silver film and silicon nitride layer with a thiol-tethered single-stranded DNA functional layer in contact with a phosphate-buffered saline medium. On the other hand, Figure 1b presents the Sys_5_ configuration, which builds on Sys_3_ by incorporating a tungsten disulfide layer between the silicon nitride and single-stranded DNA layers.

### 2.2. Modeling Approach

A numerical analysis is employed to calculate the reflectance curve using the transfer matrix method (TMM) and Fresnel equations, as described in [23,24,27]. Then, the transfer matrix describes the relationship between the tangential components of the electric and magnetic fields as:

(1) $$\left[\begin{array}{c} E_{1}\\ H_{1} \end{array}\right]=M\left[\begin{array}{c} E_{N-1}\\ H_{N-1} \end{array}\right]$$

where $E_{1}$, $H_{1}$, $E_{N-1}$, and $H_{N-1}$ represent the tangential components of electric and magnetic fields at the first and last layer interfaces, respectively. M is represented by elements $M_{ij}$ as:

(2) $$M=\overset{N-1}{\underset{k=2}{\prod }}M_{k}=\left[\begin{array}{cc} M_{11} & M_{12}\\ M_{21} & M_{22} \end{array}\right]$$

And $M_{k}$ is defined as:

(3) $$M_{k}=\left[\begin{array}{cc} \cos\beta_{k} & -i\sin\beta_{k}/q_{k}\\ -iq_{k}\;\sin\beta_{k} & \cos\beta_{k} \end{array}\right]$$

Here, k is an integer number. Additionally, $\beta_{k}$ is the phase thickness, and $q_{k}$ is the refractive index in each layer:

(4) $$\beta_{k}=\frac{2\pi d_{k}}{\lambda_{0}}\sqrt{\varepsilon_{k}-n_{1}^{2}\sin^{2}\theta }$$

And

(5) $$q_{k}=\frac{\sqrt{\varepsilon_{k}-n_{1}^{2}\sin^{2}\theta }}{\varepsilon_{k}}$$

where θ is the angle of incidence, $\lambda_{0}$ is the incident wavelength light, $n_{1}$ is the refractive index of the prism, $d_{k}$ is the thickness layer, and the local dielectric function $\varepsilon \left(\lambda_{0}\right)$ can be adopted as $n\left(\lambda_{0}\right)$. Hence, the total reflection analysis of the N-layer system is obtained as:

(6) $$R={\left|\frac{\left(M_{11}+M_{12}q_{N}\right)q_{1}-\left(M_{21}+M_{22}q_{N}\right)}{\left(M_{11}+M_{12}q_{N}\right)q_{1}+\left(M_{21}+M_{22}q_{N}\right)}\right|}^{2}$$

By using Equation (6), the SPR curve as a function of the angle of incidence is calculated. Analyzing the performance of the biosensor is necessary to consider the following metrics, defined as: the sensitivity of the biosensors (S) is denoted as the multiplication of the sensitivity to the refractive index change ($S_{RI}$) and the adsorption efficiency of the target analyte (E) as:

(7) $$S=S_{RI}\cdot E$$

For biosensor optimization, we focus on the sensitivity enhancement ($\Delta S_{RI}$) by optimizing each layer in water and PBS solutions, denoted as:

(8) $$\Delta S_{RI}=(S_{RI}^{PBS}-S_{RI}^{0})/S_{RI}^{0}$$

The sensitivity to the refractive index change can be expressed as:

(9) $$S_{RI}=\Delta \theta /\Delta n$$

The parameter Δθ represents the angle shift variation, and Δn is the change in refractive index. The detection accuracy (DA) can be written in terms of Δθ and FWHM as:

(10) DA=Δθ/FWHM

Quality factor (QF) can be expressed in terms of S and FWHM as:

(11) QF=S/FWHM

In addition, to calculate the Figure of Merit (FoM), Limit of Detection (LoD), and Signal-to-Noise ratio (SNR), the related equations can be expressed as:

(12) FoM=QF/Rmin

(13) $$\mathrm{LoD}=\frac{\Delta n}{\Delta \theta }\times 0.005$$

(14) $$\mathrm{SNR}=\frac{\Delta \theta }{\mathrm{FWHM}}$$

where Rmin is the resonance minimum from SPR curve and 0.005 is expressed in degree (0.005°). All computations in this study were performed using a data-sampling density of 20,000 points. This sampling was chosen to ensure statistical accuracy and minimize numerical errors.

## 3. Results

### 3.1. Most Promising SPR Biosensor Configurations

**Figure 2:**
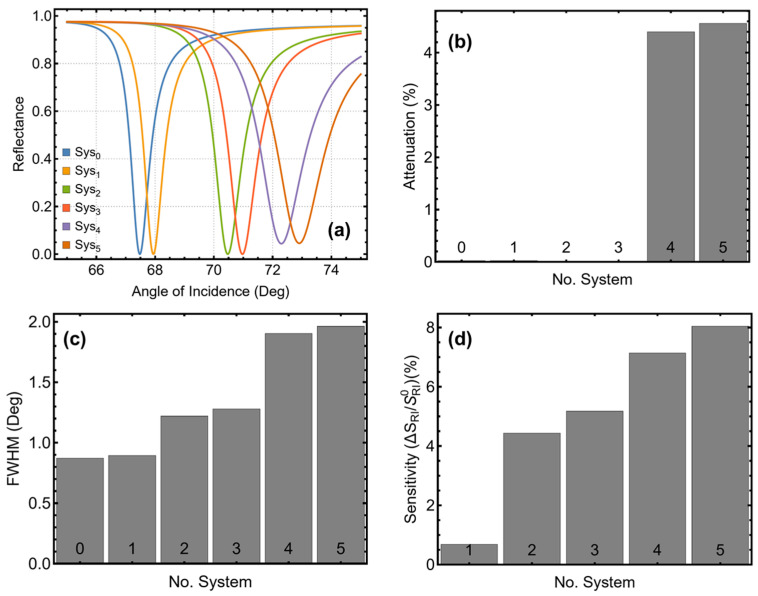
Analysis of the systems under consideration. (**a**) SPR curve as a function of the angle of incidence. Optimization metrics: (**b**) attenuation percentage, (**c**) FWHM, and (**d**) enhancement in sensitivity. Sys_0_ represents the baseline sensor with the initial parameters in water (Table S1), and the Sys_1_-Sys_5_ configurations are placed in PBS.

Figure 2 and Table S2 summarize the performance of all configurations (Sys_0_ to Sys_5_) in terms of SPR peak position (Figure 2a), attenuation (Figure 2b), FWHM (Figure 2c), and sensitivity enhancement (Figure 2d). This systematic analysis aims to identify the most promising designs for SPR biosensors by evaluating trade-offs between sensitivity enhancement and attenuation while considering peak sharpness. Sys_0_ exhibited the sharpest resonance with an FWHM of 0.87° and a minimal attenuation of 0.02%. However, its sensitivity enhancement was negligible, as expected from a simple design lacking functional or dielectric layers. Sys_1_, which replaces the water medium with PBS, showed similar results, with an FWHM of 0.90° and a marginal sensitivity enhancement of 0.68%. The inclusion of a silicon nitride layer in Sys_2_ marked a turning point in performance. This configuration shifted the SPR peak position from 67.94° to 70.47°, reflecting the increased optical interaction depth. Sys_2_ also achieved a significant sensitivity enhancement of 4.44%, although this improvement came at the cost of a slightly broader resonance with an FWHM of 1.22°. These results highlight the role of silicon nitride in stabilizing the silver layer and amplifying the plasmonic field.

Sys_3_, which incorporates a ssDNA functional layer, further increased the SPR peak position to 70.97° and achieved a sensitivity enhancement of 5.17%. Although this configuration exhibited a broader resonance (FWHM of 1.28°) compared to Sys_2_, it maintained low attenuation (0.01%). The addition of the functional layer introduces biomolecular specificity while preserving adequate signal sharpness and sensitivity, making Sys_3_ an attractive candidate for further exploration. The integration of tungsten disulfide in Sys_4_ and Sys_5_ significantly enhanced the sensitivity. Sys_4_ shifted the SPR peak position to 72.29° and achieved a sensitivity enhancement of 7.14%, while Sys_5_, which includes both tungsten disulfide and the ssDNA layer, reached an SPR peak position of 72.91° with a sensitivity enhancement of 8.04%. These configurations, however, exhibited increased attenuation (4.39% and 4.56%, respectively) and broader resonance curves, with FWHM values of 1.90° and 1.96°. Despite these trade-offs, the higher sensitivity makes Sys_4_ and Sys_5_ particularly promising.

However, Sys_3_ and Sys_5_ were primarily selected as the most promising configurations due to the inclusion of the ssDNA layer, which is critical for detecting SARS-CoV-2 RNA. The ssDNA layer provides the necessary molecular specificity by enabling hybridization with complementary RNA sequences, making these configurations highly relevant for biosensing applications targeting the virus.

### 3.2. Ag Layer Optimization

**Figure 3:**
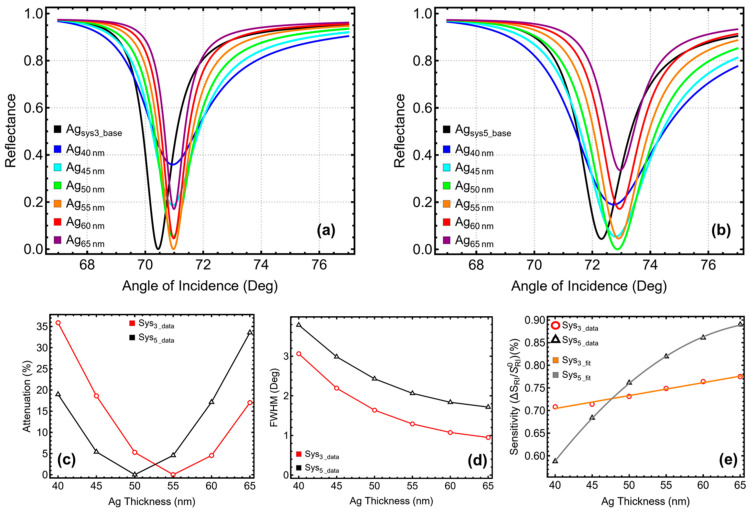
Silver optimization by increasing the layer thickness. SPR curve as a function of the angle of incidence for (**a**) Sys_3_ and (**b**) Sys_5_. Optimization metrics: (**c**) attenuation percentage, (**d**) FWHM, and (**e**) enhancement in sensitivity. Ag_Sys3_base_ and Ag_Sys5_base_ represent the baseline sensors in water (Table S1), and the Ag_40 nm_–Ag_65 nm_ configurations are placed in PBS.

The optimization of the silver layer thickness for Sys_3_ and Sys_5_ was conducted to identify configurations that minimize attenuation (Figure 3a–c) while maintaining reasonable values of FWHM (Figure 3d) and sensitivity enhancement (Figure 3e). The silver layer thickness was varied between 40 nm and 65 nm. The results, summarized in Figure 3 and Table S3, reveal distinct performance trends for both configurations, highlighting the interplay between signal sharpness, energy loss, and sensitivity enhancement.

For Sys_3_, increasing the silver thickness significantly influenced the metrics. At 40 nm, the system exhibited a high attenuation of 35.89% and a broad resonance with an FWHM of 3.06°. These results indicate that a thinner silver layer is insufficient for effective plasmonic coupling, leading to excessive energy dissipation. However, as the silver thickness increased to 55 nm, the attenuation was drastically reduced to 0.01%, while the FWHM narrowed to 1.29°, reflecting a sharper and more efficient resonance. At this thickness, Sys_3_ achieved a sensitivity enhancement of 0.75%, representing a balanced trade-off between signal clarity and sensitivity. Beyond 55 nm, further increases in thickness (e.g., 60 nm and 65 nm) caused attenuation to rise again (4.54% and 17.03%, respectively), with only marginal improvements in sensitivity enhancement (0.76% and 0.77%) and FWHM narrowing slightly to 0.95° at 65 nm. These results confirm 55 nm as the optimal thickness for Sys_3_, offering the lowest attenuation, acceptable resonance sharpness, and adequate sensitivity enhancement for practical use.

In Sys_5_, which incorporates tungsten disulfide for increased sensitivity, the optimization trends followed a similar pattern, although with some notable differences due to the additional layer. At 40 nm, the system showed attenuation of 18.97%, with an FWHM of 3.79° and a sensitivity enhancement of 0.59%. While the attenuation was lower than that of Sys_3_ at the same thickness, the resonance remained broad, limiting its practical applicability. Increasing the thickness to 50 nm reduced attenuation to 0.01% and improved the FWHM to 2.43°, while the sensitivity enhancement increased to 0.76%. This thickness provided the best compromise for Sys_5_, achieving minimal signal loss while maintaining sufficient sensitivity. Further increases in thickness to 55 nm and 60 nm slightly improved sensitivity enhancement to 0.82% and 0.86%, respectively, but introduced higher attenuation (4.56% and 17.14%). At 65 nm, the attenuation rose sharply to 33.56%, with only a slight increase in sensitivity enhancement to 0.89%. These results emphasize that 50 nm is the most suitable thickness for Sys_5_, balancing minimal attenuation, acceptable resonance sharpness, and sufficient sensitivity.

It is observed that the sensitivity enhancement exhibits a linear trend in Sys_3_ (Figure 3e, orange curve fit), whereas in Sys5, it follows a non-linear trend, approaching quasi-saturation at a thickness of 60 nm (Figure 3e, gray curve fit).

### 3.3. Silicon Nitride Layer Optimization

**Figure 4:**
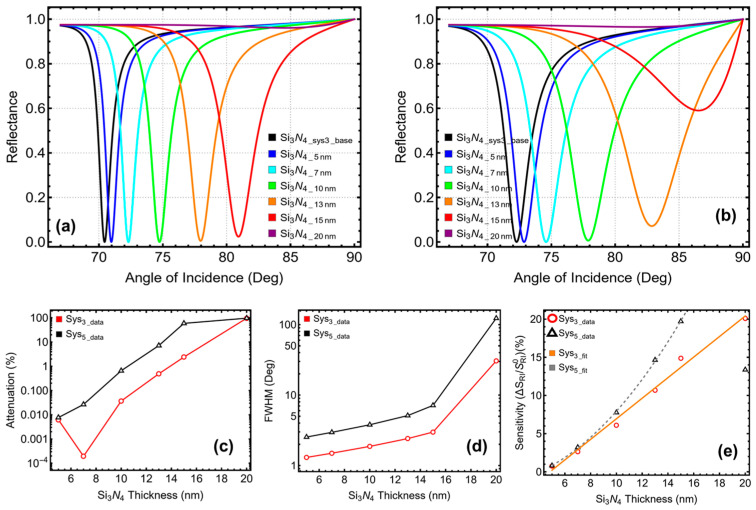
Silicon nitride optimization by increasing the layer thickness. SPR curve as a function of the angle of incidence for (**a**) Sys_3_ and (**b**) Sys_5_. Optimization metrics: (**c**) attenuation percentage, (**d**) FWHM, and (**e**) enhancement in sensitivity. Si_3_N_4_Sys3_base_ and Si_3_N_4_Sys5_base_ represent the baseline sensors in water (Table S1), and the Si_3_N_4_5 nm_–Si_3_N_4_20 nm_ configurations are placed in PBS.

Following the selection of optimal silver thicknesses, the next step was to optimize the silicon nitride layer thickness for both configurations. The results of this optimization, presented in Figure 4 and Table S4, show the influence of varying silicon nitride thicknesses (5 nm to 20 nm) on attenuation (Figure 4a–c), FWHM (Figure 4d), and sensitivity enhancement (Figure 4e). For both configurations, the selection of optimal thicknesses prioritized sensitivity enhancement while maintaining attenuation below 1% and balancing FWHM.

For Sys_3_, thinner silicon nitride layers (e.g., 5 nm) exhibited low attenuation (0.01%) and a sharp resonance (FWHM = 1.30°), but the sensitivity enhancement was limited to 0.75%. Increasing the thickness to 7 nm improved sensitivity enhancement to 2.65%, although it caused a slight broadening of the resonance peak (FWHM = 1.50°). At 10 nm, the sensitivity enhancement increased significantly to 6.10%, with an FWHM of 1.87° and attenuation remaining low at 0.04%. However, the most balanced performance was observed at 13 nm, where sensitivity enhancement reached 10.68% while maintaining a reasonable FWHM of 2.41° and acceptable attenuation of 0.49%. Beyond 13 nm, further increases in thickness caused sharp degradation in performance, with attenuation exceeding 2.40% at 15 nm and reaching an impractical 96.11% at 20 nm. These results highlight 13 nm as the optimal silicon nitride thickness for Sys_3_, achieving high sensitivity enhancement while keeping attenuation below 1% and maintaining manageable resonance broadening.

For Sys_5_, the trends followed a similar pattern, though the presence of tungsten disulfide influenced the behavior of the system. At 5 nm, Sys_5_ exhibited low attenuation (0.01%) and a relatively sharp resonance (FWHM = 2.54°), but sensitivity enhancement was limited to 0.80%. Increasing the thickness to 7 nm improved sensitivity enhancement to 3.20%, while maintaining attenuation at 0.03% and producing a slight broadening of the resonance (FWHM = 2.96°). At 10 nm, Sys_5_ achieved a significant sensitivity enhancement of 7.76%, with an FWHM of 3.79° and attenuation remaining below 1% (0.65%). Beyond 10 nm, the performance began to degrade. At 13 nm, although sensitivity enhancement increased to 14.65%, attenuation rose sharply to 7.15% and the resonance broadened significantly (FWHM = 5.11°). At thicknesses of 15 nm and 20 nm, the system exhibited excessive attenuation (59.01% and 96.51%, respectively) and impractically broad resonance peaks. These results confirm that 10 nm is the optimal silicon nitride thickness for Sys_5_, balancing high sensitivity enhancement with minimal attenuation and acceptable FWHM.

The sensitivity enhancement in Sys_3_ exhibits a linear trend, as indicated by the orange curve fit in Figure 4e. In Sys_5_, the sensitivity follows a quadratic trend up to a thickness of 15 nm (gray curve fit), beyond which a sharp decrease in sensitivity is observed at 20 nm.

### 3.4. Tungsten Disulfide Layer Optimization

**Figure 5:**
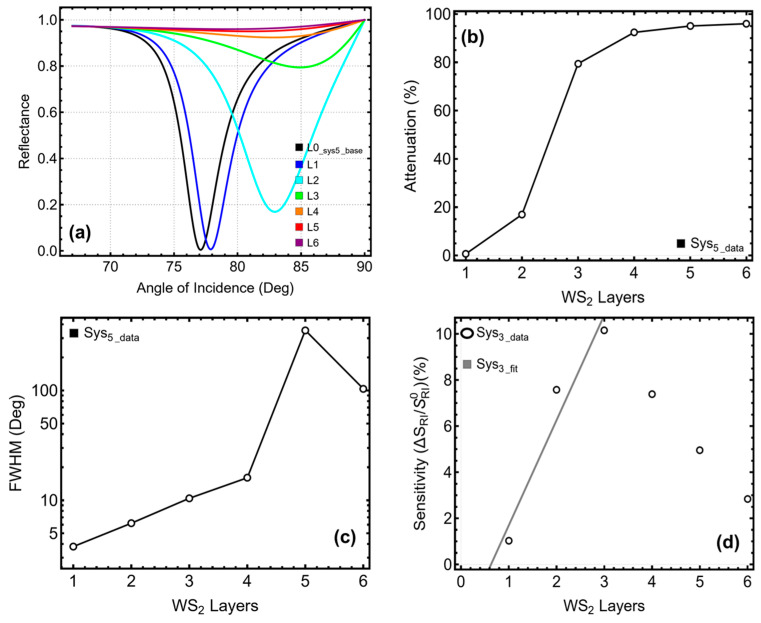
Tungsten Disulfide optimization by increasing the number of layers. (**a**) SPR curve as a function of the angle of incidence. Optimization metrics: (**b**) attenuation percentage, (**c**) FWHM, and (**d**) enhancement in sensitivity. L0__Sys5_base_ represents the baseline sensor in water (Table S1), and the L1–L6 configurations are placed in PBS.

The optimization of WS_2_ layers for the SPR biosensor demonstrates a trade-off between attenuation, FWHM, and sensitivity enhancement by analyzing configurations with one to six WS_2_ layers (Figure 5 and Table S5). The attenuation percentage (Figure 5b) shows a significant increase with the number of WS_2_ layers. It begins at 0.65% for one layer (L1) and rises steeply to 95.97% for six layers (L6). This progression highlights how additional layers intensify optical absorption, which, while beneficial for certain performance aspects, compromises the transmitted signal quality. Practical biosensing requires maintaining attenuation within acceptable limits to preserve signal strength.

The FWHM (Figure 5c) also increases progressively from 3.79° for L1 to 16.04° for L4. However, at L5, the FWHM broadens drastically to 153.15°, indicating a severe loss in resolution. Although it decreases slightly to 103.69° at L6, the general broadening remains excessive for achieving precise resonance. Sensitivity enhancement (Figure 5d) peaks at L2, reaching 7.57%, compared to 1.03% for L1. Beyond L2, sensitivity decreases progressively, with values of 10.16%, 7.39%, 4.95%, and 2.84% for L3 through L6, respectively. While configurations with additional layers yield minor improvements in sensitivity, they come at the cost of increased attenuation and FWHM broadening, reducing their practicality. Hence, considering these metrics, two WS_2_ layers (L2) represent the optimal configuration. This setup provides a substantial gain in sensitivity while keeping attenuation below 20%, maintaining a balance between performance and signal quality. Configurations with more layers lead to diminishing returns in sensitivity, coupled with severe attenuation and degraded FWHM.

### 3.5. Thiol-Tethered ssDNA Layer Optimization

**Figure 6:**
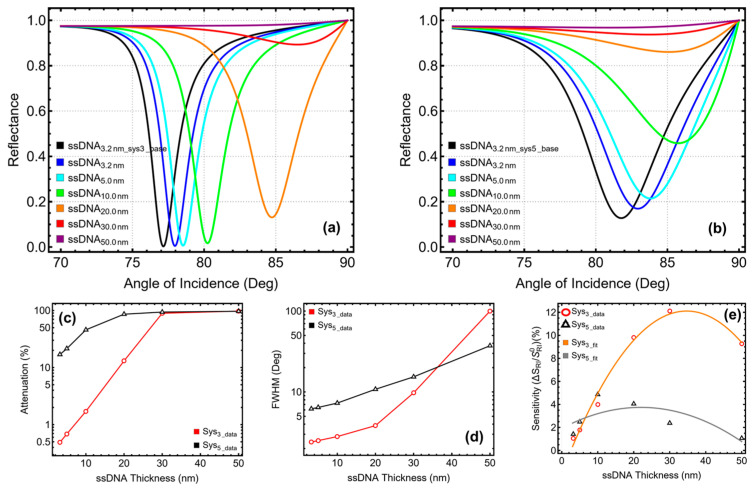
ssDNA optimization by increasing the layer thickness. SPR curve as a function of the angle of incidence for (**a**) Sys_3_ and (**b**) Sys_5_. Optimization metrics: (**c**) attenuation percentage, (**d**) FWHM, and (**e**) enhancement in sensitivity. ssDNA_3.2 nm_Sys3_base_ and ssDNA_3.2 nm_Sys5_base_ represent the baseline sensors in water (Table S1), and the ssDNA_3.2 nm_–ssDNA_50 nm_ configurations are placed in PBS.

The final step in optimizing the multilayer configurations focused on varying the thickness of the ssDNA functional layer, a critical component for ensuring biomolecular specificity in the detection of SARS-CoV-2 RNA. The optimization results, shown in Figure 6 and Table S6, evaluate the performance of Sys3 and Sys5 with ssDNA thicknesses ranging from 3.2 nm to 50 nm, considering attenuation (Figure 6a–c), FWHM (Figure 6d), and sensitivity enhancement (Figure 6e).

For Sys_3_, the initial thickness of 3.2 nm exhibited low attenuation (0.49%) and a sharp resonance with an FWHM of 2.41°. However, sensitivity enhancement at this thickness was limited to 1.05%, indicating that a slightly thicker ssDNA layer was needed to amplify the interaction between the plasmonic field and the target molecules. Increasing the thickness to 5 nm improved sensitivity enhancement to 1.78%, with a minor increase in attenuation (0.69%) and an FWHM of 2.51°. At 10 nm, Sys_3_ achieved a significant improvement, with sensitivity enhancement reaching 3.99% while keeping attenuation low at 1.71% and maintaining a reasonable FWHM of 2.82°. Beyond 10 nm, performance began to decline. At 20 nm, although sensitivity enhancement increased to 9.82%, attenuation rose sharply to 13.10% and the FWHM broadened to 3.84°, reducing the practicality of this configuration. Further increases to 30 nm and 50 nm resulted in excessive attenuation (89.31% and 97.47%, respectively) and severely broadened resonance peaks, with FWHM values of 11.23° and 50°. These results confirmed that 10 nm is the optimal ssDNA thickness for Sys_3_, balancing strong sensitivity enhancement with low attenuation and acceptable resonance sharpness.

For Sys_5_, the trends were similar, although the presence of tungsten disulfide affected the system’s response. At 3.2 nm, Sys_5_ demonstrated moderate attenuation (16.93%) and an FWHM of 3.24°, but sensitivity enhancement was limited to 1.42%. Increasing the thickness to 5 nm slightly improved sensitivity enhancement to 2.48%, with a rise in attenuation to 21.43% and a broader resonance peak (FWHM = 3.34°). At 10 nm, Sys_5_ achieved its best balance of performance metrics, with a sensitivity enhancement of 4.86%, an attenuation of 45.80%, and an FWHM of 3.62°. This configuration provided an improvement in sensitivity while maintaining manageable losses and acceptable resonance characteristics. As with Sys3, further increases in ssDNA thickness led to diminishing returns for Sys_5_. At 20 nm, sensitivity enhancement dropped slightly to 4.05%, while attenuation rose sharply to 86.06% and the FWHM broadened further to 4.26°. At 30 nm and 50 nm, the system experienced drastic degradation, with attenuation exceeding 93% and resonance peaks broadening to 5.08° and 10.34°, respectively, making these configurations impractical. These results confirmed 10 nm as the optimal ssDNA thickness for Sys_5_, balancing strong sensitivity enhancement with manageable attenuation and resonance broadening.

The sensitivity enhancement in Sys_3_ follows a polynomial trend, as demonstrated by the orange curve fit in Figure 6e. Similarly, Sys_5_ exhibits a comparable polynomial behavior, depicted by the gray curve fit. This trend contrasts with the patterns observed during the optimization of the previous layers, highlighting the unique influence of the ssDNA thickness on the overall sensitivity of the biosensors.

### 3.6. Optimized SPR Biosensors Against SARS-CoV-2

Table S7 summarizes the optimized parameters for Sys_3_ and Sys_5_, highlighting the specific thicknesses and refractive index values used for each layer. The Sys_3_ configuration, optimized with a 55 nm silver layer, a 13 nm silicon nitride layer, and a 10 nm ssDNA layer, provides a robust framework for SPR-based sensing. Similarly, Sys_5_, which incorporates tungsten disulfide with a thickness of 1.6 nm (L = 2), a 50 nm silver layer, 10 nm silicon nitride, and 10 nm ssDNA, leverages the synergistic interaction between its components to achieve high sensitivity, particularly at low analyte concentrations.

While evaluating these configurations against SARS-CoV-2, it is essential to address the refractive index values of the viral medium. Previous studies, such as those reported in [22], utilized refractive index values that are not representative of clinically viable conditions. Instead, we use the refractive index values reported in [18] as a base to obtain lower viral concentrations by applying a linear fit based on the observed data, which aligns with viral concentrations of approximately ~10^9^ particles/mL. These values provide a realistic representation of the diagnostic conditions for SARS-CoV-2 detection, bridging the gap between theoretical modeling and practical application. We point out that the inclusion of a 10 nm ssDNA layer in both configurations ensures high specificity for SARS-CoV-2 RNA detection.

**Figure 7:**
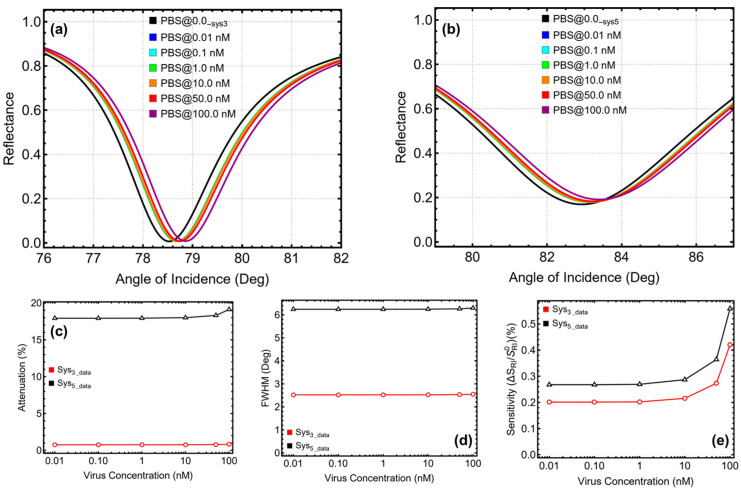
Detection analysis by increasing viral concentration. SPR curve as a function of the angle of incidence for (**a**) Sys_3_ and (**b**) Sys_5_. Optimization metrics: (**c**) attenuation percentage, (**d**) FWHM, and (**e**) enhancement in sensitivity. PBS@0.0__Sys3_ and PBS@0.0__Sys5_ represent the baseline optimized sensors in PBS, and the PBS@0.01 nM-PBS@100 nM configurations are placed in PBS+SARS-CoV-2.

Then, the optimized Sys_3_ and Sys_5_ configurations were evaluated against SARS-CoV-2 across viral concentrations ranging from 0.01 nM (~10^9^ particles/mL) to 100 nM (~10^13^ particles/mL). For each concentration, the corresponding refractive index of the medium, as shown in Table S8, was used to simulate realistic diagnostic conditions. The analysis focused on the SPR peak position (Figure 7a,b), attenuation percentage (Figure 7c), FWHM (Figure 7d), and sensitivity enhancement (Figure 7e).

For Sys_3_, the SPR peak position showed a gradual but stable shift from 78.69° at 0.1 nM to 78.86° at 100 nM, reflecting its ability to detect incremental refractive index changes induced by SARS-CoV-2. This stability in peak position is indicative of a robust plasmonic response that is not significantly affected by noise or interference. Attenuation remained remarkably low throughout the concentration range, starting at 0.75% for 0.1 nM and increasing slightly to 0.83% at 100 nM. Such minimal attenuation ensures that the signal remains strong and reliable across all tested concentrations, a key attribute for accurate biosensing. The FWHM of Sys_3_ also remained consistent, ranging between 2.52° and 2.55°, indicating sharp and well-defined resonance peaks regardless of viral load. This stability in FWHM ensures that the sensor maintains high resolution and precision, even at higher concentrations. Sensitivity enhancement increased steadily with viral concentration, starting at 0.20% for 0.1 nM and reaching 0.42% at 100 nM.

For Sys_5_, the performance metrics reflected the influence of the tungsten disulfide layer, which enhances plasmonic field confinement and sensitivity. The SPR peak position shifted more significantly compared to Sys_3_, increasing from 83.16° at 0.1 nM to 83.40° at 100 nM. However, this increased sensitivity comes with higher attenuation values, starting at 17.93% for 0.1 nM and rising to 19.11% at 100 nM. While higher attenuation may reduce signal intensity, the trade-off is justified by the system’s enhanced sensitivity. The FWHM for Sys_5_ remained broader than Sys_3_, with values ranging from 6.24° to 6.30°. This broader resonance indicates a slightly lower precision compared to Sys_3_, though it is compensated by the superior sensitivity enhancement of Sys_5_. Starting at 0.26% for 0.1 nM, sensitivity enhancement increased steadily to 0.55% at 100 nM, demonstrating a strong capability of Sys_5_ to amplify the plasmonic response even at higher viral concentrations.

### 3.7. Performance Metrics

**Figure 8:**
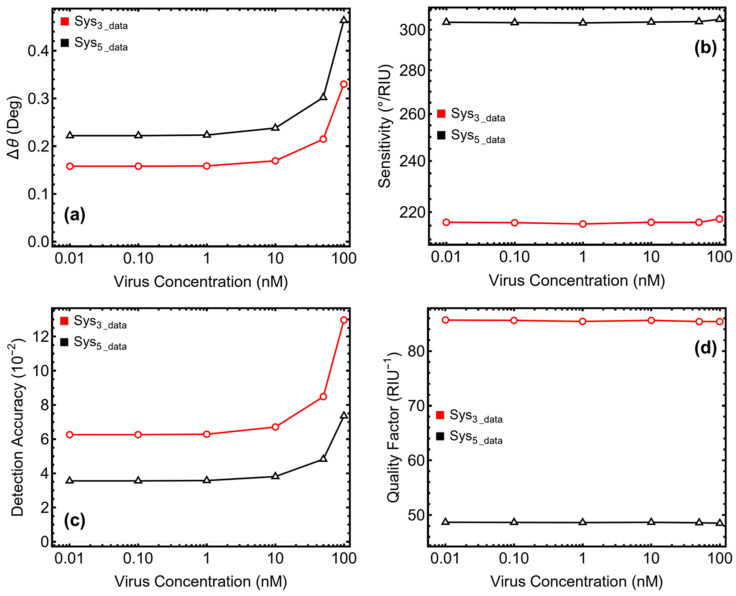
Performance metrics of the optimized biosensors Sys_3_ and Sys_5_: (**a**) angle variation, (**b**) sensitivity to refractive index change, (**c**) detection accuracy, and (**d**) quality factor.

The performance of the optimized Sys_3_ and Sys_5_ biosensors was analyzed in terms of angle variation (Δ*θ*, Figure 8a), sensitivity to refractive index change (*S*, Figure 8b), detection accuracy (DA, Figure 8c), and quality factor (QF, Figure 8d) (see Table 2). The angle variation reflects the shift in the SPR resonance angle caused by changes in refractive index due to the presence of SARS-CoV-2. For Sys_3_, Δ*θ* increased gradually from 0.158° at 0.1 nM to 0.33° at 100 nM, showing a stable response to incremental changes in viral concentration. This steady increase highlights the ability of Sys_3_ to provide consistent angular shifts, which is critical for applications that require precise quantification of analyte concentrations. In comparison, Sys_5_ exhibited a more pronounced increase in Δ*θ*, ranging from 0.22° at 0.1 nM to 0.46° at 100 nM. The larger angle shifts observed in Sys_5_ are attributed to the enhanced plasmonic field confinement introduced by the tungsten disulfide layer, which amplifies its sensitivity to refractive index changes.

Sensitivity, expressed in degrees per refractive index unit (°/RIU), quantifies the biosensors’ ability to detect refractive index changes. Sys_3_ demonstrated moderate but stable sensitivity values, starting at 216.10 °/RIU for 0.1 nM and increasing slightly to 217.47 °/RIU at 100 nM. This stability underscores the robustness and reliability of Sys_3_ in handling varying viral concentrations. On the other hand, Sys_5_ exhibited consistently higher sensitivity, ranging from 303.63 °/RIU at 0.1 nM to 305.33 °/RIU at 100 nM. The heightened sensitivity of Sys_5_ stems from the presence of tungsten disulfide, which enhances plasmonic interactions, making it highly effective for detecting small refractive index variations.

Detection accuracy measures the precision of the biosensors in identifying refractive index changes. Sys_3_ exhibited a significant improvement in DA as the viral concentration increased, starting at 0.04 × 10^−2^ for 0.1 nM and rising to 48.93 × 10^−2^ at 100 nM. This consistent improvement highlights the ability of Sys_3_ to deliver precise detection, particularly at higher viral concentrations. In contrast, Sys_5_ demonstrated lower DA values, starting at 0.02 × 10^−2^ at 0.1 nM and increasing to 28.85 × 10^−2^ at 100 nM. The broader resonance peaks in Sys_5_ contribute to its lower DA compared to Sys_3_. However, its higher sensitivity ensures that it remains effective for detecting subtle refractive index changes at low concentrations, despite the reduced precision.

The quality factor, which evaluates the sharpness and energy efficiency of the resonance, further differentiates the two configurations. Sys_3_ maintained consistently high QF values, starting at 86.87 RIU^−1^ for 0.1 nM and remaining above 81 RIU^−1^ across all concentrations. These high values reflect the ability of Sys_3_ to maintain sharp and well-defined resonance peaks, crucial for applications requiring precise signal clarity. In comparison, Sys_5_ exhibited lower QF values, starting at 45.84 RIU^−1^ for 0.1 nM and slightly decreasing to 48.08 RIU^−1^ for 100 nM. The lower QF for Sys_5_ is a result of its broader resonance peaks, which, while enhancing sensitivity, reduce resonance sharpness and energy efficiency.

**Figure 9:**
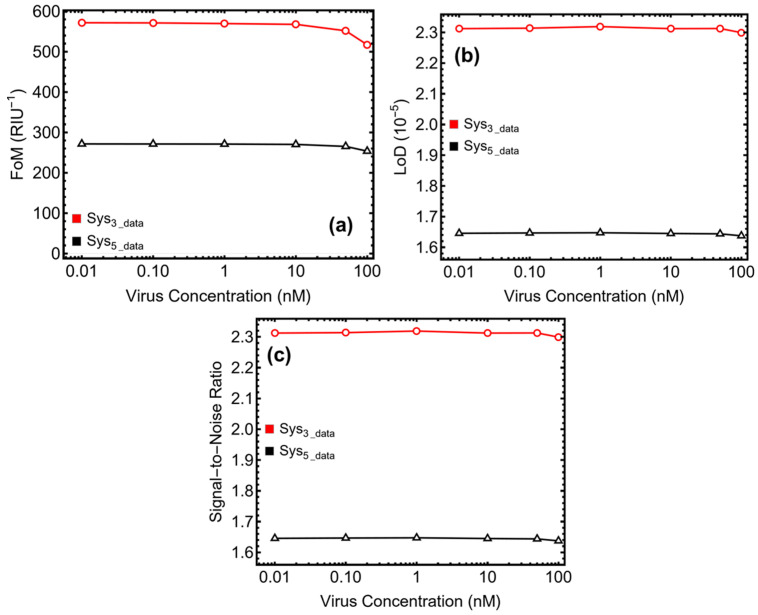
Performance metrics of the optimized biosensors Sys_3_ and Sys_5_: (**a**) figure of merit, (**b**) limit of detection, and (**c**) signal-to-noise ratio.

The performance evaluation of Sys_3_ and Sys_5_ was concluded by analyzing their figure of merit (FoM, Figure 9a), limit of detection (LoD, Figure 9b), and signal-to-noise ratio (SNR, Figure 9c). The results, summarized in Figure 9 and Table 3, highlight the distinct strengths and trade-offs of each configuration.

The FoM, which measures the balance between sensitivity and FWHM, remained consistently high for Sys_3_. Starting at 571.24 RIU^−1^ at 0.1 nM, the FoM showed a gradual decrease to 517.12 RIU^−1^ at 100 nM. This stability highlights the facility of Sys_3_ to maintain sharp resonance peaks while responding effectively to changes in refractive index. The slight decline at higher concentrations can be attributed to minor resonance broadening but remains within acceptable limits for precise sensing. Sys_5_, on the other hand, exhibited lower FoM values across all concentrations, starting at 271.28 RIU^−1^ at 0.1 nM and dropping to 253.60 RIU^−1^ at 100 nM. The reduced FoM is a result of the broader resonance peaks in Sys_5_, which trade off sharpness for higher sensitivity. Despite this, the FoM values for Sys_5_ remain adequate for detecting subtle refractive index changes.

The LoD provides critical insight into the biosensors’ ability to detect low analyte concentrations. Sys_3_ maintained a stable LoD of approximately 2.31 × 10^−5^ across the tested range, demonstrating its capability for detecting SARS-CoV-2 at concentrations as low as 0.01 nM. This low LoD reflects a high precision and suitability of Sys_3_ for early diagnostic applications. Sys_5_, while achieving slightly higher LoD values than Sys_3_, consistently performed at approximately 1.65 × 10^−5^. This enhanced sensitivity, attributed to the tungsten disulfide layer, highlights the utility of Sys_5_ in detecting very low viral loads where sensitivity is the priority.

The SNR, which measures the clarity of the signal relative to background noise, was consistently higher for Sys_3_ compared to Sys_5_. For Sys_3_, the SNR started at 0.06 at 0.1 nM and increased to 0.12 at 100 nM, demonstrating its ability to generate clear, distinguishable signals across the concentration range. This reliability makes Sys_3_ particularly suited for precision diagnostics. In contrast, Sys_5_ exhibited lower SNR values, beginning at 0.04 at 0.1 nM and peaking at 0.07 at 100 nM. The reduced SNR for Sys_5_ is a consequence of higher attenuation and broader resonance peaks, which introduce more background noise. Despite this limitation, the SNR observed in Sys_5_ remains sufficient for reliable detection, especially in applications where sensitivity is more critical than signal clarity.

### 3.8. Literature Comparison with Related SPR Biosensor Architectures

Table 4 compares the performance of our proposed SPR biosensors, Sys_3_ and Sys_5_, with recent configurations reported in the limited literature on this topic [28,29,30,31,32]. While these works provide valuable insights into SPR biosensor development, it is important to highlight that they do not specifically focus on SARS-CoV-2 detection or clinically relevant concentration ranges. Furthermore, none of these studies present a comprehensive analysis of performance metrics, such as sensitivity, quality factor, detection accuracy, figure of merit, limit of detection, and signal-to-noise ratio, as performed in our work. This broader evaluation accentuates the novelty and thoroughness of our approach. Lin et al. [28] reported a hybrid structure combining graphene, MoS_2_, WSe_2_, and WS_2_, achieving a sensitivity of 194 °/RIU. In comparison, our Sys_3_ and Sys_5_ configurations deliver superior sensitivities of 217.5 °/RIU and 305.3 °/RIU, respectively, at clinically relevant viral concentrations (0.01–100 nM). Bijalwan et al. [29] investigated nanoribbons of graphene and WSe_2_, achieving a sensitivity of 155.68 °/RIU and a QF of 164.28 RIU^−1^. Sys_3_ and Sys_5_ outperform this sensitivity, with Sys_5_ achieving a QF of 50.4 RIU^−1^ at 1 nM. These findings emphasize the potential of our biosensors to address the stringent requirements of SARS-CoV-2 detection at early stages. Dey et al. [30] combined WS_2_, metal layers, and graphene to achieve a sensitivity of 208 °/RIU and a QF of 223.66 RIU^−1^. Mostufa et al. [31] proposed a multilayer design with BK7, WS_2_, Au, BaTiO_3_, and graphene, achieving a sensitivity of 230.77 °/RIU. While comparable to Sys_3_, their study does not address specific viral detection scenarios. In contrast, our results demonstrate that Sys_5_ offers not only higher sensitivity but also a detailed evaluation of performance metrics across a realistic concentration range. Rahman et al. [32] presented a biosensor based on Au-WS_2_-PtSe_2_-BP, reporting a sensitivity of 200 °/RIU and a QF of 17.70 RIU^−1^. While their QF is relatively low, their study does not specify the concentration range, making it challenging to assess its diagnostic utility.

## 4. Conclusions

In this study, we developed and optimized two multilayered SPR biosensors, namely Sys_3_ and Sys_5_, for detecting SARS-CoV-2 across clinically relevant concentrations. By systematically analyzing and optimizing each biosensor layer, including silver, silicon nitride, and ssDNA, we achieved configurations tailored for high sensitivity and diagnostic reliability. Sys_3_, incorporating a 55 nm silver layer, a 13 nm silicon nitride layer, and a 10 nm ssDNA layer, demonstrated exceptional precision with low attenuation, sharp resonance peaks, and high detection accuracy. Sys_5_, enhanced with a 50 nm silver layer, a 10 nm silicon nitride layer, a 10 nm ssDNA layer, and bilayer tungsten disulfide (L = 2 = 1.6 nm), showed superior sensitivity and a lower limit of detection, making it particularly suitable for identifying low analyte concentrations.

Key findings revealed the strengths of Sys_3_ in maintaining sharp resonance and energy efficiency, reflected by its high figure of merit (FoM), low attenuation, and high signal-to-noise ratio (SNR). Sys_5_, on the other hand, outshined in sensitivity to refractive index changes and demonstrated a consistent ability to detect subtle variations in refractive index, even at the lowest viral concentrations. Both configurations exhibited excellent alignment with the refractive index ranges of SARS-CoV-2 clinical samples, evidencing their potential applicability for realistic diagnostic scenarios. Moreover, the refractive index values used in this study were adapted from clinically viable ranges, overcoming the limitations of previous works that relied on less representative values.

This work not only emphasizes the diagnostic potential of these biosensors but also highlights the importance of systematic optimization in SPR-based sensing systems. The combination of physical and performance metrics, including angle variation, sensitivity, detection accuracy, and quality factor, provided a robust framework for evaluating biosensor performance under realistic operating conditions.

## Figures and Tables

**Table 1:** Systems under configurations considering different stacking layer order.

Sys No.	Code	Full Name	Notation
0	Sys_0_	Prism/Silver/Water medium	P/Ag/M_H2O_
1	Sys_1_	Prism/Silver/PBS medium	P/Ag/M_PBS_
2	Sys_2_	Prism/Silver/Si_3_N_4_/PBS medium	P/Ag/SN/M_PBS_
3	Sys_3_	Prism/Silver/Si_3_N_4_/ssDNA/PBS medium	P/Ag/SN/T/M_PBS_
4	Sys_4_	Prism/Silver/Si_3_N_4_/Tungsten Disulfide/PBS medium	P/Ag/SN/WS_2_/M_PBS_
5	Sys_5_	Prism/Silver/Si_3_N_4_/Tungsten Disulfide/ssDNA/PBS medium	P/Ag/SN/WS_2_/T/M_PBS_

**Table 2:** Performance metrics of optimized Sys_3_ and Sys_5_ configurations, including the angle variation, sensitivity, detection accuracy, and quality factor.

Concentration (mM)	RI: PBS + SARS-CoV-2	Δθ	S (°/RIU)	DA (10^−2^)	QF (*RIU*^−1^)
**Sys_3_**					
0.01	1.3347306664113630	0.0	0.0	0.0	0.0
0.1	1.3347311385238432	0.158	216.10	0.04	86.87
1.0	1.3347358596486474	0.159	215.62	0.37	91.14
10	1.3347830708966875	0.169	216.24	3.73	93.35
50	1.3349928986657549	0.214	216.20	18.59	92.94
100	1.3355174680884236	0.33	217.47	48.93	81.56
**Sys_5_**					
0.01	1.3347306664113630	0.0	0.0	0.0	0.0
0.1	1.3347311385238432	0.22	303.63	0.02	45.84
1.0	1.3347358596486474	0.22	303.5	0.20	50.41
10	1.3347830708966875	0.24	303.93	1.94	48.46
50	1.3349928986657549	0.30	304.16	9.67	48.35
150	1.3355174680884236	0.46	305.33	28.85	48.08

**Table 3:** Performance metrics of optimized Sys_3_ and Sys_5_ configurations, including the figure of merit, limit of detection, and signal-to-noise ratio.

Concentration (mM)	RI: PBS + SARS-CoV-2	FoM (*RIU*^−1^)	LoD (10^−5^)	SNR
**Sys_3_**				
0.01	1.3347306664113630	0.0	0.0	0.0
0.1	1.3347311385238432	571.24	2.31	0.06
1.0	1.3347358596486474	569.6	2.32	0.06
10	1.3347830708966875	567.63	2.31	0.07
50	1.3349928986657549	551.71	2.31	0.08
150	1.3355174680884236	517.12	2.29	0.12
**Sys_5_**				
0.01	1.3347306664113630	0.0	0.0	0.0
0.1	1.3347311385238432	271.28	1.65	0.04
1.0	1.3347358596486474	271.04	1.65	0.04
10	1.3347830708966875	270.26	1.65	0.04
50	1.3349928986657549	265.31	1.64	0.05
150	1.3355174680884236	253.60	1.64	0.07

**Table 4:** Comparison of the maximum sensing performance of the proposed SPR biosensors with recently reported configurations, including sensitivity, concentration range, and quality factor.

Ref.	Configuration	Sensitivity (°/RIU)	Concentration Testing	QF (RIU^−1^)
Lin et al., 2020 [28]	Hybrid Structure with Graphene/MoS_2_/WSe_2_/WS_2_	194	---	---
Bijalwan et al., 2020 [29]	Nanoribbons of Graphene and WSe_2_	155.68	---	164.28
Dey et al., 2021 [30]	WS_2_/Metal/WS_2_/Graphene	208	---	223.66
Mostufa et al., 2022 [31]	BK7/WS_2_/Au/BaTiO_3_/Graphene	230.77	---	---
Rahman et al., 2022 [32]	Au-WS_2_-PtSe_2_-BP	200	---	17.70
This work	Sys_3_ Sys_5_	217.5 @ 100 nM 305.3 @ 100 nM	0.01–100 (nM)	93.4 @ 10 nM 50.4 @ 1 nM

## Data Availability

The original contributions presented in the study are included in the article/Supplementary Materials. Further inquiries can be directed to the corresponding author.

## Supplementary Materials

The following supporting information can be downloaded at: https://www.mdpi.com/article/10.3390/mi16020128/s1, Table S1: Initial parameters of the SPR Biosensor under investigation by reporting the corresponding refractive index at 633 nm; Table S2: Analysis of physical and performance metrics of the different configurations; Table S3: Analysis of physical and performance metrics of Sys_3_ and Sys_5_ configurations by changing the silver thickness; Table S4: Analysis of physical and performance metrics of Sys_3_ and Sys_5_ configurations by changing the silicon nitride thickness; Table S5: Analysis of physical and performance metrics of Sys_5_ configuration by changing the number of Tungsten Disulfide layers; Table S6: Analysis of physical and performance metrics of Sys_3_ and Sys_5_ configurations by changing the ssDNA thickness; Table S7: Optimized parameters of Sys_3_ and Sys_5_ configurations; Table S8: Analysis of performance metrics of Sys_3_ and Sys_5_ configurations at different virus concentrations.
