# Supplementary material for: WS2/Si3N4-Based Biosensor for Low-Concentration Coronavirus Detection
Source: Micromachines (Basel). 2025 Jan 23;16(2):128. doi: 10.3390/mi16020128 (PMC11857482; doi:10.3390/mi16020128)
Supplement: Supplementary file 1 [file micromachines-16-00128-s001.zip › micromachines-3426336-supplementary.pdf]

micromachines

SUPPLEMENTARY INFORMATION: WS_2/Si_3N_4-Based Biosensor for Low-Concentration Coronavirus Detection

Talia Tene [1,*], Fabian Arias Arias [2], Karina I. Paredes-Páliz [3], Ana M. Cunachi Pillajo [4], Ana Gabriela Flores Huilcapi [2], Luis Santiago Carrera Almendariz [5] and Stefano Bellucci [6]

[1] Department of Chemistry, Universidad Técnica Particular de Loja, Loja 110160, Ecuador
[2] Dipartimento di Chimica e Tecnologie Chimiche, University of Calabria, Via P. Bucci, Cubo 15D, 87036 Rende, Italy
[3] Grupo de Investigación en Salud Pública, Facultad de Ciencias de la Salud, Universidad Nacional de Chimborazo, Riobamba 060108, Ecuador
[4] Laboratorio Ciencias Biológicas, Facultad Recursos Naturales, Escuela Superior Politécnica de Chimborazo (ESPOCH), Riobamba 060155, Ecuador
[5] Facultad de Ciencias, Escuela Superior Politécnica de Chimborazo (ESPOCH), Riobamba 060155, Ecuador
[6] INFN-Laboratori Nazionali di Frascati, Via E. Fermi 54, 00044 Frascati, Italy

* Correspondence: tbtene@utpl.edu.ec

Supplementary Tables

Table S1. Initial parameters of the SPR Biosensor under investigation by reporting the corresponding refractive index at 633 nm

Material	Refractive Index at 633 nm	Thickness (nm)	Ref.
BK-7 (P)	1.5151	---	[21]
Silver (Ag)	0.056253 + 4.2760 i	55.0	[24]
Si_3N_4 (SN)	2.0394	5.00	[22]
Tungsten Disulfide (WS_2)	4.9 + 0.3124 i	0.80	[18]
ssDNA (Thiol-Tethered, T)	1.462	3.20	[22]
Water medium (H_2O)	1.33	---	[23]
PBS medium	1.334	---	[24]

Table S2. Analysis of physical and performance metrics of the different configurations

Sys No.	Code	SPR Peak position	Attenuation (%)	FWHM	Enhancement (%)
0	Sys_0	67.48	0.02	0.87	0.00
1	Sys_1	67.94	0.02	0.90	0.68
2	Sys_2	70.47	0.01	1.22	4.44
3	Sys_3	70.97	0.01	1.28	5.17
4	Sys_4	72.29	4.39	1.90	7.14
5	Sys_5	72.91	4.56	1.96	8.04

Table S3. Analysis of physical and performance metrics of Sys_3 and Sys_5 configurations by changing the silver thickness

**Thickness (nm)**	**SPR Peak position**	**Attenuation (%)**	**FWHM**	**Enhancement (%)**
		**Sys_3**		
40	70.94	35.89	3.06	0.71
45	70.95	18.61	2.19	0.71
50	70.96	5.27	1.64	0.73
55	70.97	0.01	1.29	0.75
60	70.98	4.54	1.07	0.76
65	70.99	17.03	0.95	0.77
		**Sys_5**		
40	72.74	18.97	3.79	0.59
45	72.81	5.39	2.98	0.68
50	72.86	0.01	2.43	0.76
55	72.91	4.56	2.06	0.82
60	72.94	17.14	1.84	0.86
65	72.96	33.56	1.72	0.89

Table S4. Analysis of physical and performance metrics of Sys_3 and Sys_5 configurations by changing the silicon nitride thickness

Thickness (nm)	SPR Peak position	Attenuation (%)	FWHM	Enhancement (%)
**Sys_3**				
5	70.97	0.01	1.30	0.75
7	72.31	0.0001	1.50	2.65
10	74.74	0.04	1.87	6.10
13	77.97	0.49	2.41	10.68
15	80.93	2.40	2.99	14.88
20	84.60	96.11	30.61	20.10
**Sys_5**				
5	72.86	0.01	2.54	0.80
7	74.59	0.03	2.96	3.20
10	77.89	0.65	3.79	7.76
13	82.86	7.15	5.11	14.65
15	86.52	59.01	7.13	19.71
20	81.94	96.51	123.261	13.37

Table S5. Analysis of physical and performance metrics of Sys_5 configuration by changing the number of Tungsten Disulfide layers

Layers	SPR Peak position	Attenuation (%)	FWHM	Enhancement (%)
L1	77.89	0.65	3.79	1.03
L2	82.93	16.92	6.18	7.57
L3	84.92	79.42	10.43	10.16
L4	82.79	92.38	16.04	7.39
L5	80.92	95.03	153.15	4.95
L6	79.29	95.97	103.69	2.84

Table S6. Analysis of physical and performance metrics of Sys_3 and Sys_5 configurations by changing the ssDNA thickness

Thickness (nm)	SPR Peak position	Attenuation %	FWHM	Enhancement (%)
		**Sys_3**		
3.2	77.97	0.49	2.41	1.05
5.0	78.53	0.69	2.51	1.78
10.0	80.24	1.71	2.82	3.99
20.0	84.73	13.10	3.84	9.82
30.0	86.51	89.31	11.23	12.12
50.0	70.0	97.47	50.0	9.11
		**Sys_5**		
3.2	82.94	16.93	3.24	1.42
5.0	83.81	21.43	3.34	2.48
10.0	85.75	45.80	3.62	4.86
20.0	85.08	86.06	4.26	4.05
30.0	83.72	93.73	5.08	2.38
50.0	80.90	96.79	10.34	1.21

Table S7. Optimized parameters of Sys_3 and Sys_5 configurations

**Material**	**Refractive Index (RI)**	**Thickness (nm)**
	**Sys_3**	
BK7 (P)	1.5151	---
Ag	0.056253 + 4.2760	55.0
Si_3N_4 (SN)	2.0394	13.0
ssDNA (Thiol-Tethered, T)	1.462	10.00
PBS (M)	1.334	---
	**Sys_5**	
BK7 (P)	1.5151	---
Ag	0.056253 + 4.2760	50.0
Si_3N_4 (SN)	2.0394	10.0
Tungsten Disulfide	4.9 + 0.3124 i	0.80*2 (L=2)
ssDNA (Thiol-Tethered, T)	1.462	10.0
PBS (M)	1.334	---

Table S8. Analysis of performance metrics of Sys_3 and Sys_5 configurations at different virus concentrations

Concentration (mM)	RI: PBS + SARS-CoV-2	SPR Peak position	Attenuation %	FWHM	Enhancement (%)
**Sys_3**					
0.01	1.3347306664113630	0.0	0.0	0.0	0.0
0.1	1.3347311385238432	78.69	0.75	2.52	0.20
1.0	1.3347358596486474	78.69	0.75	2.52	0.20
10	1.3347830708966875	78.70	0.75	2.53	0.21
50	1.3349928986657549	78.75	0.77	2.53	0.27
100	1.3355174680884236	78.86	0.83	2.55	0.42
**Sys_5**					
0.01	1.3347306664113630	0.0	0.0	0.0	0.0
0.1	1.3347311385238432	83.16	17.93	6.24	0.26
1.0	1.3347358596486474	83.16	17.93	6.24	0.27
10	1.3347830708966875	83.17	18.00	6.24	0.29
50	1.3349928986657549	83.23	18.31	6.26	0.36
100	1.3355174680884236	83.40	19.11	6.30	0.55
